# Massive Hematochezia from a Large Bleeding Duodenal Diverticulum

**DOI:** 10.1155/2021/5585264

**Published:** 2021-06-08

**Authors:** Marcus Juan Esteban, Amit Sureen, Daniel Herlihy, Sherif Elhanafi, Marc J. Zuckerman

**Affiliations:** ^1^Division of Gastroenterology, Texas Tech University Health Sciences Center, El Paso, TX, USA; ^2^Department of Internal Medicine, Texas Tech University Health Sciences Center, El Paso, TX, USA

## Abstract

**Background:**

Duodenal diverticula are a rare cause of gastrointestinal (GI) bleeding despite being a common finding in the GI tract. We present a case of a patient who had massive hematochezia due to a complex duodenal diverticulum. *Case Presentation.* A 74-year-old Hispanic female presented initially with generalized weakness. During admission, the patient had an episode of a large amount of hematochezia and had to be transferred to the intensive care unit (ICU). Upper endoscopy was done using a forward-viewing endoscope which revealed a bleeding complex duodenal diverticulum. Successful hemostasis was achieved through epinephrine injection followed by placement of hemostatic clips.

**Conclusion:**

Although rare, gastroenterologists need to be aware of duodenal diverticulum as a possible cause of gastrointestinal bleed. It could be life-threatening, and thus, prompt diagnosis and management is necessary.

## 1. Introduction

Diverticula are common findings in the adult GI tract and are mostly found in the colon [1]. The duodenum is the second most common location for GI tract diverticula [2]. Duodenal diverticula are often asymptomatic [1–3]. Possible complications include mechanical obstruction of the duodenum, biliary tract or pancreatic duct, inflammation causing perforation, abscess and fistula formation, and hemorrhage [1, 2]. We present a rare case of a patient who had massive hematochezia due to a complex duodenal diverticulum. Successful hemostasis was achieved with epinephrine injection followed by placement of hemostatic clips.

## 2. Case

**Figure 1:**
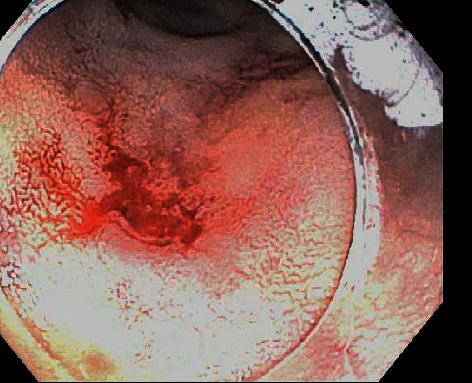
Actively oozing angiodysplasias within the diverticulum.

**Figure 2:**
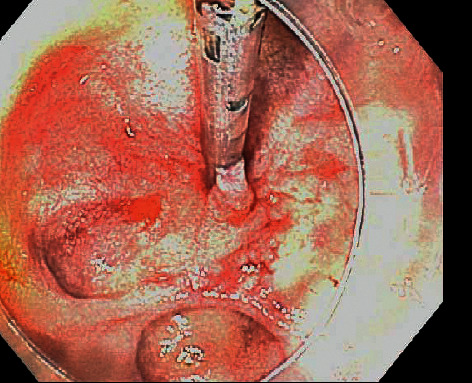
Angiodysplasias after injection and hemostatic clip placement. No bleeding was seen postintervention.

A 74-year-old Hispanic female with a past medical history of type 2 diabetes mellitus and hypertension presented with generalized weakness. She initially denied any gastrointestinal bleeding. She had no history of aspirin or NSAID use and was not on any anticoagulants. On admission, vital signs were normal and she had pale conjunctiva. Her abdomen was mildly tender to palpation. Laboratory findings were significant for Hgb 6.7 g/dL and blood glucose of 494 mg/dL. INR was 1.0 on admission. She was admitted for transfusion and blood sugar management. After admission, she had an episode of a large amount of bright red blood per rectum. She became hypotensive and Hgb dropped to 3.7 g/dL. She was intubated, transferred to the intensive care unit, and started on continuous pantoprazole infusion. Gastric lavage gave a clear aspirate. Repeat Hgb was 10.5 g/dL after transfusion with 4 units of packed RBCs. Esophagogastroduodenoscopy (EGD) using a forward-viewing endoscope was done which showed a 30 mm diverticulum in the area of the major papilla with multiple smaller diverticula within the main diverticulum. There were several angiodysplasias within the diverticulum that were oozing blood (Figure 1). The lesions were injected with epinephrine (1 : 10,000 dilution) and four hemostatic clips were placed (Figure 2). There was no further bleeding after the intervention. Hemoglobin remained stable throughout the rest of admission and she was eventually discharged home. The patient had no gastrointestinal issues at the 3-month follow-up.

## 3. Discussion

Diverticula are common findings in the adult GI tract and are found predominantly in the large colon [1]. The duodenum is the second most common location for GI tract diverticula after the colon [2]. In a retrospective review of patients over 23 years, 208 patients were identified with small bowel diverticula. Of these, 79% were in the duodenum, 18% were in the jejunum or ileum, and 3% were in the duodenum, jejunum, and ileum [4]. The precise incidence of duodenal diverticula is unknown, but based on autopsy data, it ranges from 3% to 22%. Radiologic findings on the upper gastrointestinal series estimate it at 2% to 5% [3, 5]. In a large series of 624 patients who underwent endoscopic retrograde cholangiopancreatography, the reported incidence was 23% [6]. There are two types of duodenal diverticula: endoluminal and extraluminal. The latter type is the most frequent. Endoluminal diverticula, as in our patient's case, are caused by congenital webs of the second duodenum. Extraluminal diverticula are known to be associated with bile duct disorders [3].

Duodenal diverticula are often asymptomatic [1–3]. Complications that have been reported include mechanical obstruction of the biliary tract causing obstructive jaundice and cholangitis, mechanical obstruction of the pancreatic duct causing pancreatitis, mechanical obstruction of the duodenum, inflammation causing perforation, abscess and fistula formation, and hemorrhage, which can be occult or profuse [1, 2]. The exact incidence of hemorrhage from duodenal diverticula is unknown [7, 8]. In the abovementioned retrospective study of 208 patients with small bowel diverticula, bleeding complications were reported in 14 patients [6] Diverticular bleeding can be due to an inflamed diverticulum, erosion of a diverticulum into a major vessel, arteriovenous malformations within the diverticulum, aortoenteric fistula formation, or angiodysplasia [6]. Less common causes of bleeding duodenal diverticula include Dieulafoy lesions in the diverticulum and bleeding secondary to intradiverticular polyps [9, 10]. Bleeding due to diverticular angiodysplasia, such as in our case, is exceptionally rare [5].

Given its rarity, a bleeding duodenal diverticulum may be unsuspected and can be difficult to diagnose. Diagnosis has been facilitated with the advent of endoscopy [11]. Upper endoscopy is the preferred modality of diagnosis for bleeding duodenal diverticulum as it also provides the opportunity to perform therapeutic interventions. Upper endoscopy may fail to reveal diverticula in the fourth portion of the duodenum, but it is recommended that the examination of the duodenum be extended as far as possible when the indication for endoscopy is gastrointestinal hemorrhage [3, 11]. In our case, we were fortunate to locate the bleeding source with a forward-viewing scope. In some cases, however, a side-viewing duodenoscope may be necessary [5, 11, 12]. Diverticulectomy previously was the primary treatment for the duodenal diverticulum [3, 13]. With the advances in endoscopy, various endoscopic therapeutic strategies such as injection with epinephrine, bipolar coagulation, argon plasma coagulation (APC), and hemostatic clips have been used for the treatment of bleeding duodenal diverticula [5, 9, 11, 12, 14]. In our case, successful hemostasis was achieved with epinephrine injection followed by placement of hemostatic clips. Surveillance endoscopy is not indicated for bleeding duodenal diverticulum.

## 4. Conclusion

Duodenal diverticula should be included in the consideration of rare causes of hematochezia. Prompt diagnosis and treatment is necessary to avoid life-threatening complications as was demonstrated in our case.
